# Increased mRNA Levels of *ADAM17*, *IFITM3*, and *IFNE* in Peripheral Blood Cells Are Present in Patients with Obesity and May Predict Severe COVID-19 Evolution

**DOI:** 10.3390/biomedicines10082007

**Published:** 2022-08-18

**Authors:** Catalina A. Pomar, M. Luisa Bonet, Adrián Ferre-Beltrán, Pablo A. Fraile-Ribot, Mercedes García-Gasalla, Melchor Riera, Catalina Picó, Andreu Palou

**Affiliations:** 1 Laboratory of Molecular Biology, Nutrition and Biotechnology, Group of Nutrigenomics, Biomarkers and Risk Evaluation, University of the Balearic Islands, 07122 Palma, Spain; 2 Health Research Institute of the Balearic Islands (IdISBa), 07120 Palma, Spain; 3 Centro de Investigación Biomédica en Red (CIBER) de Fisiopatología de la Obesidad y Nutrición, Instituto de Salud Carlos III, 28029 Madrid, Spain; 4 Sección de Enfermedades Infecciosas de Medicina Interna, Hospital Universitario Son Espases (HUSE), 07120 Palma, Spain; 5 Servicio de Microbiologia, Hospital Universitario Son Espases (HUSE), 07120 Palma, Spain

**Keywords:** COVID-19, transcriptomic biomarkers, peripheral blood cells

## Abstract

Gene expression patterns in blood cells from SARS-CoV-2 infected individuals with different clinical phenotypes and body mass index (BMI) could help to identify possible early prognosis factors for COVID-19. We recruited patients with COVID-19 admitted in Hospital Universitari Son Espases (HUSE) between March 2020 and November 2021, and control subjects. Peripheral blood cells (PBCs) and plasma samples were obtained on hospital admission. Gene expression of candidate transcriptomic biomarkers in PBCs were compared based on the patients’ clinical status (mild, severe and critical) and BMI range (normal weight, overweight, and obesity). mRNA levels of *ADAM17*, *IFITM3*, *IL6*, *CXCL10*, *CXCL11*, *IFNG* and *TYK2* were increased in PBCs of COVID-19 patients (*n* = 73) compared with controls (*n* = 47), independently of sex. Increased expression of *IFNE* was observed in the male patients only. PBC mRNA levels of *ADAM17*, *IFITM3*, *CXCL11*, and *CCR2* were higher in those patients that experienced a more serious evolution during hospitalization. *ADAM17,* *IFITM3*, *IL6* and *IFNE* were more highly expressed in PBCs of patients with obesity. Interestingly, the expression pattern of *ADAM17*, *IFITM3* and *IFNE* in PBCs was related to both the severity of COVID-19 evolution and obesity status, especially in the male patients. In conclusion, gene expression in PBCs can be useful for the prognosis of COVID-19 evolution.

## 1. Introduction

The coronavirus disease 2019 (COVID-19) pandemic caused by the betacoronavirus SARS-CoV-2 represents the main public health, social and economic problem since the 2nd world war. Although reported COVID-19 deaths between 1 January 2020, and 31 December 2021, totaled 5.94 million worldwide, a more realistic figure based on excess mortality is that 18.2 million people died worldwide over that period because of COVID-19 [1]. The more than half a billion reported cases result in diverse rates of mortality depending on differences in many influencing factors, including health conditions that put individuals at high risk of getting seriously ill [2].

The clinical spectrum of COVID-19 ranges from asymptomatic, mild to moderate, severe, and critical disease [3]. Obesity or excess ectopic fat deposition is a prominent risk factor for a more serious and complicated course of the disease [4,5], as substantiated by various metanalysis [6,7]. The prognosis of COVID-19 is consistently associated to worsen with obesity but also with various conditions including older age, male sex, and comorbidities such as diabetes, hypertension, chronic respiratory disease and cardiovascular disease, that are strongly related to obesity and, particularly, to dysfunctional visceral adipose tissue [2]. Visceral fat is considered to be particularly detrimental because it produces higher amounts of proinflammatory cytokines that are released into the bloodstream and can lead to auto-amplifying cytokine production, the so called “cytokine storm” that seems to mediate the progression to COVID-19 critical illness [8]. A general assumption is that, in individuals with obesity, obesity-related or derived metabolic dysfunctions—including alterations in insulin, leptin, and adiponectin homeostasis, and loss of control of other adipokines—combined with cardiorespiratory dysfunction and immune dysregulation to mediate the progression to critical illness and/or diminished treatments effectivity [5]. However, beyond general considerations, precise components of the mechanisms responsible for greater COVID-19 severity in persons with obesity remain largely unknown. Mechanistic understanding of the relationship between obesity and COVID-19 is highly relevant because it may suggest more accurate therapeutic interventions.

Peripheral blood cells (PBCs) and peripheral blood mononuclear cells (PBMCs) are increasingly being used for clinical and nutritional studies [9,10,11,12,13] because these cells can be easily collected in sufficient quantities, stored frozen for years, and, most importantly, they can reflect gene expression patterns of key physiological and pathological responses and conditions of the organism [12,14], including diseases or alterations related to metabolism [9,12,15,16] and the immune system [11]. PBCs can collect information from the tissues through which they circulate, so that gene expression in these cells is affected by signals that typically reach and affect other parts of the body, and it changes in response to both internal and external clues [9,12,15]. Recently, it was reported that obesity-related visceral adipose tissue shows hypermethylation and downregulation of the *ACE2* (angiotensin-converting enzyme 2) gene, which encodes the key receptor for the entry of SARS-CoV-2 into the human cells [17]. Interestingly, the methylation and expression pattern of the *ACE2* gene was mirrored in PBMCs and restored after nutritional weight reduction therapy [17].

SARS-CoV-2 entry into cells is mediated by the viral S (spike) protein, located at the virus membrane [18]. The entry involves virus binding to ACE2 receptor at the cell surface followed by fusion to deliver the uncoated viral-RNA into the cell for its transcription and replication. The fusion step is dependent on target-cell proteases that activate fusion by cleaving S protein subunit 2 (S2) at an internal site. A major route occurs at the plasma membrane and involves S2 cleavage by type II transmembrane serine protease (TMPRSS2), present at the cell surface. Another route is TMPRSS2-independent and involves virus endocytosis followed by cathepsin-mediated cleavage and activation of S2 for membrane fusion, which occurs in the endolysosome [18]. The cellular interferon-induced antiviral transmembrane protein IFITM3 may affect the cell surface entry route but is a factor initially known to act at the endosomal entry route of different virus, including coronavirus [19]. Besides, it is known that ACE2 membrane abundance in different cell types is tightly regulated by the desintegrin and metalloproteinase domain 17 (ADAM17), whose up regulation leads to the proteolytic cleavage and release of the ACE2 ectodomain [20,21].

Since the emergence of the COVID-19 pandemic, several studies have addressed blood cell transcriptomics —in some studies as part of wider multi-omics approaches— to better understand the pathophysiology of COVID-19 and the biological bases of COVID-19 severity [22,23,24,25]. Nowadays, the disease and its mechanisms are better understood, making it feasible to focus on a subset of mechanistically important genes, without the restrictions of vast multi-testing penalties. Therefore, in this work we hypothesized that expression levels in easily obtainable blood cells of key host genes known from studies in *bona fide* virus sensitive tissues (lung, airways and related) to be involved in SARS-CoV-2 entry mechanisms and early host responses could provide solid prospective markers of the disease course. Gene expression at the mRNA level is well suited to this end, since it mainly informs about biological responses that are being prepared and about to happen-in contrast to genomic markers, which inform about predispositions, or protein or metabolic markers, which rather inform about what has already happened.

Thus, to gain further insight into COVID-19 pathophysiology, we obtained PBC samples of controls and COVID-19 patients on admission to the hospital to analyze the expression of selected genes that are part of the known ACE2-based port of entry of SARS-CoV-2 into cells (*ADAM17*, *ACE2*, *TMPRSS2* and *IFITM3*), together with other genes encoding cytokines expressed in blood cells (*IL6*, *IL7*, *IFNG*, *IFNE*, *CXCL8*, *CXCL9*, *CXCL10*, *CXCL11*), or previously reported to be related to COVID-19 severity (*CCR2*, *TYK2*, *EDN1*) [26,27]. The study was focused on those of these genes found to be expressed significantly in PBC human samples, and in comparing their patterns of expression in patients that later experienced different degrees of severity, and in patients in different body mass index (BMI) ranges, given that obesity is a main risk factor for COVID-19 severity [6,7].

## 2. Materials and Methods

### 2.1. Patients Consent Statement

The study protocol was approved by the Ethics Committee of Research of the Balearic Islands (CEI-IB) (nºIB 4360/20 PI) and was carried out in accordance with The Code of Ethics of the World Medical Association (Declaration of Helsinki). Written consent was obtained from each patient or subject after full explanation of the purpose and nature of all procedures. Until their analysis, samples were stored in the Health Research Institute of the Balearic Islands Biobank (Ref. B527 of Spanish national biobanks).

### 2.2. Participants

The study included 73 COVID-19 patients who were admitted at Hospital Son Espases (Balearic Islands, Spain) between March 2020 and July 2021. COVID-19 diagnosis was verified by the positivity of the SARS-CoV-2 PCR assay of nasopharyngeal samples. The severity of signs and symptoms developed during hospitalization was categorized as mild, severe, and critical as previously described [28].

In addition, 47 healthy volunteers, who had no symptoms, were enrolled. To be included in the study, healthy volunteers had to be free of clinically significant disease or medical conditions. The exclusion criteria were being a minor; physically or legally incapacitated person; pregnant or lactating; suffering from contagious, infectious diseases, or at risk of complications during blood collection (such as coagulation problems and heart failure); and history of alcohol or drug abuse.

### 2.3. Anthropometric Measurements

Collected anthropometric data included body weight and height. The BMI was calculated as weight (kilograms) divided by height (meters) squared. Obesity phenotypes were defined based on individual BMI as normal weight (BMI < 25), overweight (BMI = 25–30) and obesity (BMI > 30).

### 2.4. Sample Collection

At the time of hospitalization, blood samples were collected in anticoagulant free-tubes (EDTA) and in PAXgene vacutainer tubes (QIAGEN, Hilden, Germany) via antecubital fossa venipuncture. To obtain plasma for analyses of biochemical parameters, the tubes were centrifuged twice in order to obtain PPP (platelet-poor plasma). First centrifugation was at 1600× *g* for 10 min, after which plasma supernatant was transferred into a falcon tube, and a second centrifugation (2500× *g*, 10 min) was applied.

### 2.5. Real-Time qPCR Analysis in Whole Blood Cells

Total RNA was isolated using the PAXgene blood RNA kit according to the manufacturer’s instructions (QIAGEN, Hilden, Germany). RNA quality and purity were analyzed by spectrophotometry using the Nanodrop ND-1000, and RNA integrity was confirmed using agarose gel electrophoresis.

Total RNA (225 ng) was transcribed into cDNA using an iScript cDNA synthesis kit (Bio-Rad Laboratories, Madrid, Spain) in an Applied Biosystems 2720 Thermal Cycler. After cDNA synthesis, real-time qPCR was performed for each RT product to determine mRNA expression of the selected genes. The expression of ADAM metallopeptidase domain 17 (*ADAM17*; NM_003183.6), interferon induced transmembrane protein 3 (*IFITM3*; NM_021034.3), interferon epsilon (*IFNE*, NM_176891.4), interferon gamma (*IFNG*, NM_000619.3), C-X-C motif chemokine ligand 8 (*CXCL8*, NM_001354840.3), C-X-C motif chemokine ligand 9 (*CXCL9*, NM_002416.3), C-X-C motif chemokine ligand 10 (*CXCL10*, NM_001565.4), C-X-C motif chemokine ligand 11 (*CXCL11*, NM_001302123.2), interleukin 6 (*IL6*, NM_000600.5), interleukin 7 (*IL7*, NM_001199888.1), C-C motif chemokine receptor 2 (*CCR2*, NM_001123396.4), tyrosine kinase 2 (*TYK2*, NM_001385199.1), and endothelin 1 (*EDN1*, NM_001955.5) was analyzed by RT-qPCR in PBCs from all volunteers as previously described [29]. Gene expression data were normalized against the housekeeping tripartite motif containing 27 (*TRIM27*; NM_006510.5). This gene has been previously described as a stable reference gene for PCR normalization [30]. Primers sequences for the different genes are shown in Supplementary Table S1. All primers were purchased from Sigma Genosys (Sigma Aldrich Química SA, Madrid, Spain).

### 2.6. Analysis of Blood Parameters

The following circulating parameters were measured in patients by the analytical service of the Son Espases University Hospital (HUSE), using standard procedures: hemoglobin (g/dL), D-dimer (ng/mL), lactate dehydrogenase (U/L), bilirubin (mg/dL), Glutamate Pyruvate Transaminase (GPT, U/L), glutamic oxaloacetic transaminase (GOT, U/L), gamma-glutamil transpeptidase (GGT, U/L), Urea (mg/dL), Glucose (mg/dL), C-reactive protein (mg/dL), Interleukine 6 (pg/mL), ferritin (ng/mL), and Vitamin D (ng/mL).

### 2.7. Statistical Analysis

Data are expressed as mean ± standard error of the mean (SEM). The normality was checked using the Kolmogorov-Smirnov test, and the homoscedasticity among groups was assessed using Levene’s test. When one or both of these conditions were not accomplished, data were logarithm-transformed (using log base 10) to achieve a normal distribution and/or similar variances before performing statistical tests. Differences between healthy controls and COVID-19 patients were analyzed by Student’s *t* test. Differences between controls and COVID-19 patients segmented by severity (mild, severe, and critical course) and by BMI range (normal weight, overweight, and obesity) were analyzed by one-way ANOVA followed by least significant difference (LSD) post-hoc comparison. Healthy controls were not stratified by BMI range for analysis due to limited sample size in each group. Different lowercase letters (a, b, c) indicate significant differences between groups by LSD post-hoc test. Linear regression model was used to evaluate the relationship between gene expression analysis and the BMI. All statistical analyses were performed with SPSS for Windows v 21 (SPSS, Chicago, IL, USA). Threshold of significance was set at *p* <  0.05 for all analyses.

## 3. Results

### 3.1. Subject Characteristics

We collected PBCs and plasma from 47 control subjects and 73 COVID-19 patients admitted in HUSE. The subject characteristics of each group are shown in Table 1. COVID-19 patients were older than control subjects and displayed similar body weight, height, and BMI to control subjects. Of the 73 recruited patients, 33 developed mild COVID-19 during hospitalization (45.2%), 24 developed severe COVID-19 (32.9%), and 16 developed critical COVID-19 (21.9%). A tendency was observed for critical patients to present a greater body weight (*p* = 0.063, one-way ANOVA, post-hoc analysis, a-ab-b). Specifically, the body weight of the critical group was significantly higher than that of the group that developed a mild COVID-19 (*p* = 0.021, Student’s *t* test). Regarding the circulating parameters (Table 2), lactate dehydrogenase activity increased as a function of COVID-19 severity (one-way ANOVA, post-hoc analysis, a-b-c). Urea, C-reactive protein, and ferritin were similarly increased in both severe and critical patients as compared with mild COVID-19 patients (one-way ANOVA, post-hoc analysis, a-b-b), whereas GOT and GGT were significantly increased only in the severe patients (one-way ANOVA, post-hoc analysis, a-b-ab). IL-6 circulating levels tended to increase with the degree of COVID-19 severity, but differences did not reach statistical significance.

A second analysis of the anthropometric measurements and circulating parameters of the COVID-19 patients was carried out according to BMI range (Table 1 and Table 2, respectively). Of the 73 recruited patients, 20 (27.4%) were normal weight (BMI < 25), 32 (43.8%) were overweight (BMI = 25–30), and 21 (28.8%) had obesity (BMI > 30). Regarding circulating parameters, patients with obesity displayed greater hemoglobin levels (one-way ANOVA, post-hoc analysis, a-ab-b), and overweight patients lower bilirubin levels (one-way ANOVA, post-hoc analysis, a-b-ab) as compared with the normal weight patients.

### 3.2. Assessment of Sex-Specific Differences in Gene Expression of Selected Genes

**Figure 1:**
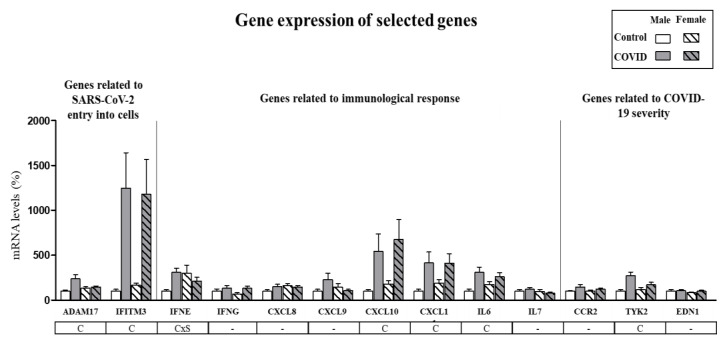
Expression levels of selected genes in PBCs in male and female control subjects and COVID-19 patients. mRNA levels were measured by real-time qPCR and expressed as a percentage of the value of control males. Data are expressed as the mean ± SEM of 15–43 samples per group. Statistics: C, effect of COVID; CxS, interaction between COVID and sex (*p* < 0.05, two-way ANOVA).

Expression of selected genes in PBCs of control subjects and COVID-19 patients was first analyzed in males and females separately, to identify possible sex-specific differences. Supplementary Table S2 shows the detailed information for all the genes analyzed (mean, standard error of the mean (SEM), confidence interval, median, minimum, and maximum). As presented in Figure 1, gene expression of *ADAM17*, *IFITM3*, *CXCL10*, *CXCL11*, *IL6* and *TYK2* was increased in PBCs of COVID-19 patients as compared with control subjects independently of sex (*p* < 0.05, two-way ANOVA). For *IFNE* gene expression an interactive effect between COVID-19 disease and sex was detected (*p* < 0.05, two-way ANOVA), so that expression was increased in male COVID-19 patients compared to their controls but not in females. These results suggest that gene expression in PBCs from men and women can be considered together for most of the analyzed genes, with the exception of *IFNE*, which should be considered separately for both sexes.

### 3.3. Expression Levels in PBCs of SARS-CoV-2 Cell Entry-Related Genes According to COVID-19 Severity and Obesity

**Figure 2:**
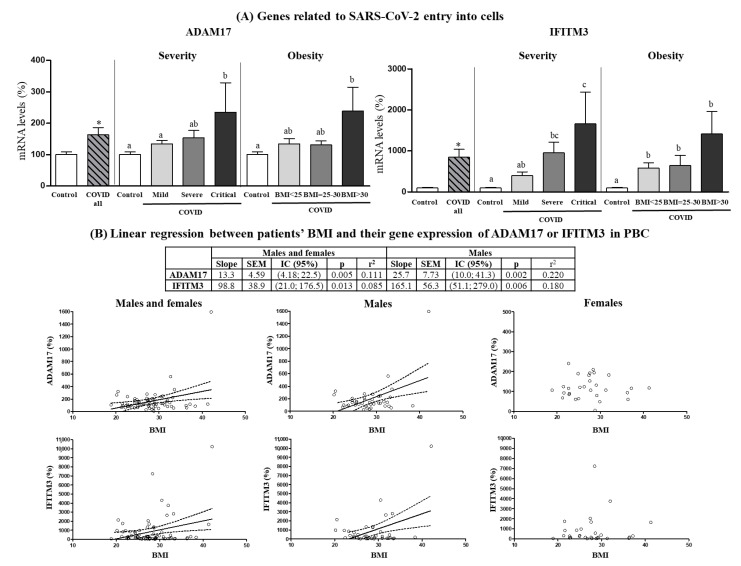
(**A**) Expression levels in PBCs of the indicated genes related to SARS-CoV-2 entry into cells in control subjects and COVID-19 patients (all) and separated according to COVID-19 severity and obesity. (**B**) Linear regression between patients’ BMI and their gene expression in PBCs of *ADAM17* or *IFITM3*. mRNA levels were measured by real-time qPCR and expressed as a percentage of the value of Controls (males and females). Data are expressed as the mean ± SEM. Statistics: differences between stratified groups were analyzed by one-way ANOVA followed by least significant difference (LSD) post-hoc test, a ≠ b ≠ c (*p* < 0.05). Student’s *t* test was used for single comparisons: *, COVID all vs Control.

Expression levels of genes related to SARS-CoV-2 entry into cells in PBCs of patients on hospital admission are shown in Figure 2A. *ACE2* and *TMPRSS2* mRNAs could not be detected in PBCs, in agreement with previous reports indicating very low expression of these two genes in PBMCs/PBCs as compared to other human tissues [31,32]. Gene expression levels of *ADAM17* and *IFITM3* were increased in PBCs of COVID-19 patients compared with control subjects (by 63% and 700%, respectively). Interestingly, *ADAM17* and *IFITM3* mRNA levels were maximal in the patients that became critically ill during hospitalization (one-way ANOVA, post-hoc analysis) and in patients with obesity (with BMI > 30) (one-way ANOVA, post-hoc analysis). These differences were especially evident in the male patients. In fact, when male and female patients were analyzed independently (Supplementary Figure S1), an increase in *ADAM17* gene expression in PBC that was grossly proportional to COVID-19 severity and BMI range was evidenced in the males, whereas in the females no significant differences were found between control subjects and COVID-19 patients, nor by COVID-19 severity, or obesity. The pattern of *IFITM3* gene expression in PBCs was similarly affected by COVID-19 and COVID-19 severity in both sexes, yet only male obese patients displayed a greater expression than overweight patients (one-way ANOVA, post-hoc analysis). For both *ADAM17* and *IFITM3*, gene expression levels in PBCs of patients were predicted by BMI, particularly in the male patients (Figure 2B), in which for each unit of increase in BMI, gene expression increased by 13.3 ± 4.59 units for *ADAM17* and by 98.8 ± 38.9 units for *IFITM3*. In the case of control subjects, although they were not stratified by BMI due to the limited sample size, mRNA levels of *ADAM17* and *IFITM3* were not predicted by BMI, neither when both sexes were pooled, nor when males and females were considered separately (data not shown).

### 3.4. Expression Levels in PBCs of Immunological Response-Related Genes According to COVID-19 Severity and Obesity

**Figure 3:**
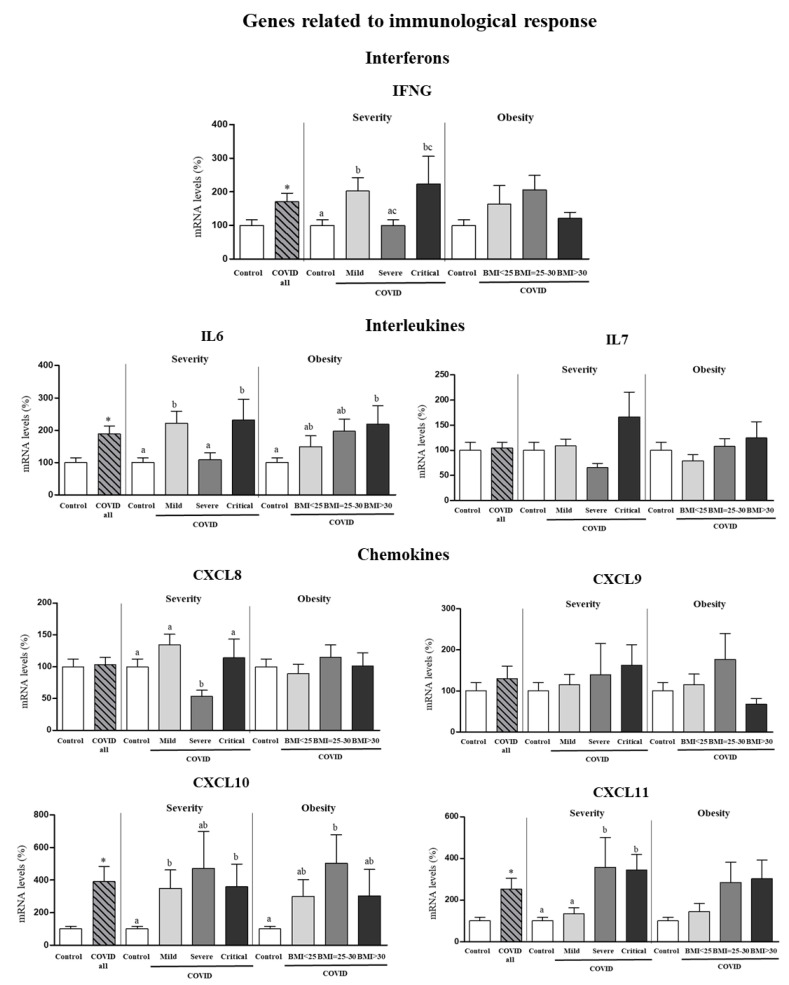
Expression levels in PBCs of the indicated genes related to immunological response in control subjects and COVID-19 patients (all) and separated according to COVID-19 severity and obesity. Pooled data from males and females are shown since no sex effects or sex × COVID interactive effects were detected for the indicated genes. mRNA levels were measured by real-time qPCR and expressed as a percentage of the value of control subjects (male and female). Data are expressed as the mean ± SEM. Statistics: differences between stratified groups were analyzed by one-way ANOVA followed by least significant difference (LSD) post-hoc test, a ≠ b ≠ c (*p* < 0.05). Student’s t test was used for single comparisons: *, COVID all vs Control.

Expression levels in PBCs of genes for interferons (*IFNG*), interleukins (*IL6* and *IL7*) and chemokines (*CXCL8, CXCL9, CXCL10, CXCL11*) whose expression was not affected by sex according to results in Figure 1 are shown as pooled data from males and females (Figure 3). Gene expression of *IFNG* was increased by 70% in PBCs of COVID-19 patients compared to control subjects (Student’s *t* test), and the increase was more marked in mild and critical patients than in severe patients (one-way ANOVA, post-hoc analysis). No differences in *IFNG* gene expression in PBCs according to BMI categories of COVID-19 patients were observed. Regarding the interleukins, gene expression of *IL6*, but not *IL7*, was increased in PBCs of COVID-19 patients (by 89%) as compared to control subjects (Student’s *t* test). Interestingly, mild and critical patients displayed greater *IL6* mRNA levels in PBCs than severe patients (one-way ANOVA, post-hoc analysis). Furthermore, when categorized by BMI, only obese patients (BMI > 30) showed greater *IL6* mRNA levels in PBCs than controls (one-way ANOVA, post-hoc analysis). Regarding the chemokines, COVID-19 patients showed gene expression levels of *CXCL8* and *CXCL9* similar to control subjects, but increased expression levels of *CXCL10* and *CXCL11* (by 290% and 150%, respectively). To be noted, increased gene expression of *CXCL11* was observed in severe and critical patients but not in patients that developed a mild COVID-19 (one-way ANOVA, post-hoc analysis). Interestingly, *CXCL11* mRNA levels tended also to be higher in obese and overweight patients as compared to normal weight patients (despite not reaching significant differences).

**Figure 4:**
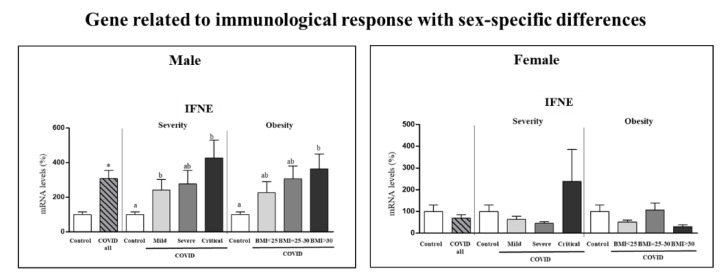
Expression levels in PBCs of the indicated gene related to immunological response in controls and COVID-19 patients (male and female) and separated according to COVID-19 severity and obesity in each sex. mRNA levels were measured by real-time qPCR and expressed as a percentage of the value of the respective control sex (male or female). Data are expressed as the mean ± SEM. Statistics: differences between stratified groups were analyzed by one-way ANOVA followed by least significant difference (LSD) post-hoc test, a ≠ b (*p* < 0.05). Student’s *t* test was used for single comparisons: *, COVID all vs Control.

Gene expression in PBCs of interferon epsilon (*IFNE*) exhibited sex-specific differences (Figure 1), and therefore it was analyzed according to COVID-19 severity and obesity independently in the two sexes (Figure 4). Differences were observed only in males. Specifically, male COVID-19 patients, but not females, had increased expression levels of *IFNE* in PBCs (by 208%) compared to control subjects (Student’s *t* test), and the increase was exacerbated in critical and obese patients, despite no significant differences among patient categories.

### 3.5. Expression Levels in PBCs of Other Genes Related to COVID-19 Severity

**Figure 5:**
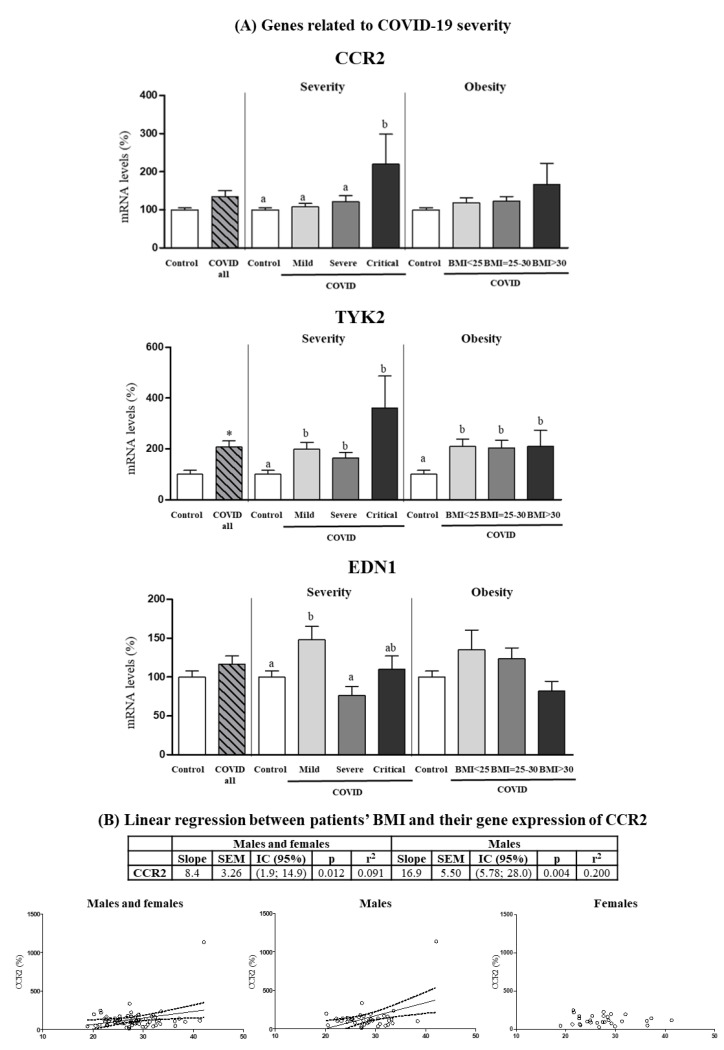
(**A**) Expression levels in PBCs of the indicated genes related to COVID severity in control subjects and COVID-19 patients (all) and separated according to COVID severity and obesity. (**B**) Linear regression between patients’ BMI and their gene expression in PBCs of *CCR2*. mRNA levels were measured by real-time qPCR and expressed as a percentage of the value of Control (male and female). Data are expressed as the mean ± SEM. Statistics: differences between stratified groups were analyzed by one-way ANOVA followed by least significant difference (LSD) post-hoc test, a ≠ b (*p* < 0.05). Student’s *t* test was used for single comparisons: *, COVID all vs Control.

*CCR2*, *EDN1* and *TYK2* genes were analyzed for mRNA expression in PBCs given their previously described relation with COVID-19 severity [22,23] (Figure 5). PBCs *CCR2* mRNA levels were similar in COVID-19 patients and control subjects when all patients were considered but were greater in critical patients as compared to controls or less severe patient groups (one-way ANOVA, post-hoc analysis). Despite no differences were observed among BMI ranges, *CCR2* gene expression levels in PBCs of patients were predicted by BMI, particularly in the male patients (Figure 5B), in which for each unit of increase in BMI, gene expression increased by 16.9 ± 5.50 units. They were also predicted by BMI in PBCs of the control subjects, albeit only when pooling both sexes and with a lower slope value (2.00 ± 0.76) (*p* = 0.012; r^2^ = 0.147).

*EDN1* mRNA levels were also similar in COVID-19 patients and control subjects when all patients were considered and, strikingly, were greater in mild patients as compared to controls or more severe patients (one-way ANOVA, post-hoc analysis). Finally, *TYK2* mRNA levels were increased (by 107%) in COVID-19 patients compared to control subjects (Student’s *t* test), and the increase was evidenced in all COVID-19 severity and BMI categories (one-way ANOVA, post-hoc analysis).

## 4. Discussion

Early prognosis factors for COVID-19 are crucial for selecting and adapting effective personalized treatment strategies. Obesity and male sex have both been associated with a poor prognosis in patients with COVID-19. Here, we show that a number of genes known to be related to the routes of entry of SARS-CoV-2 into human cells or to the host immunological response against virus infection are overexpressed in the PBCs of patients at the time of hospital admission, some of them differentially depending on the disease severity outcome developed, BMI or sex. PBCs are a type of minimally invasive samples that are easy to routinely obtain, store and handle and are able to reflect ongoing changes in body tissues, therefore of great interest as a potential source of clinically relevant markers.

*ADAM17* was substantially overexpressed in blood cells of COVID-19 patients, and more markedly in those patients that ulteriorly experienced a more critical outcome. ADAM17 is a “sheddase” metalloproteinase whose activity in cells releases the ectodomains of a number of membrane-anchored proteins, among them the receptor for SARS-CoV and SARS-CoV-2, ACE2 [20]. ADAM17 activity is enhanced by the SARS-CoV S protein, and it was reported that ADAM17-catalysed ACE2 cleavage fosters SARS-CoV entry into cells [33], although there are conflicting results [34]. Regulation of ADAM17 by the novel coronavirus SARS-CoV-2 remains largely unknown: our results suggest that SARS-CoV-2 infection enhances *ADAM17* gene expression and therefore, based on the assumption that a higher gene expression level usually translates into a higher protein amount and higher protein activity, it may result in increased ADAM17 activity.

Increased *ADAM17* gene expression in PBCs depending on COVID-19 severity outcome as observed in this work is in good concordance with previous suggestions that ADAM17 sheddase activity may play a crucial role in the pathogenesis of COVID-19 [35,36], even if a functional role in SARS-CoV-2 entry routes remains unclear [37]. ADAM17 activity may play such a crucial role by (i) decreasing the levels of intact membrane-bound ACE2 and (ii) releasing a number of proinflammatory factors through cleavage of their membrane-anchored precursor molecules. ACE2 functions physiologically to convert proinflammatory, profibrotic and vasoconstrictor Angiotensin II into Angiotensin (1–7), which antagonizes the effects of Angiotensin II [38,39]. Thus, it can be assumed that increased ADAM17 cleavage activity on ACE2 would result in increased circulating Angiotensin II levels that may contribute to the vascular pathophysiology of COVID-19 [40] and to renal and other systemic complications [41]. Moreover, ADAM17 is the primary sheddase for proinflammatory TNFalfa release from the surface of cells [42], and it acts on the IL-6 receptor (IL-6R) to release its soluble form (sIL-6R) [43]. Binding of IL-6 to the sIL-6R results in an agonistic IL-6/sIL-6R complex that activates cells (via gp130) irrespective of whether the cells express the IL-6R itself [43]. This so-called trans-signaling pathway is thought to mainly account for the pro-inflammatory properties of IL-6, and it would be exacerbated under conditions of increased both *ADAM17* expression and circulating IL-6 levels, as observed in the present study in COVID-19 patients depending on the disease severity outcome. To be noted, higher circulating levels of sIL-6R and IL6 have been associated to the exacerbated immune response against SARS-CoV-2 [43,44].

Maximal *ADAM1*7 expression in PBCs of COVID-19 patients with obesity (BMI > 30) is explicable, considering that obesity often associates with metabolic inflammation, with which ADAM17 activity has recognized links, through proteolytic processing of pro-TNFalfa, ACE2, IL-6R and other membrane-anchored proteins in adipose tissue and other tissues [45,46]. Evidence in humans and rodents indicates that aging and obesity cooperatively increase vascular endothelial ADAM17 activity and the release of soluble TNFalfa by adipose tissue cells [47]. Even a causal relationship between increased ADAM17 and obesity was suggested from the knocking out of *Adam17* in mice, which leads to an extremely lean phenotype due to hypermetabolism [48]. Metabolic inflammation associates specifically with visceral obesity, which is more common in obese men than women [49]. Interestingly, when results were analyzed by sex, increases in *ADAM17* gene expression in PBCs with both BMI range and COVID-19 severity degree were evidenced in the male patients only. Further, a direct correlation of *ADAM17* gene expression with BMI was found in male patients, but not in female patients or in control subjects, by linear regression model. Unfortunately, precise fat distribution in our patients is not available, yet the sex-specific changes observed could reflect the higher prevalence of visceral obesity in men and be in line with ADAM17 playing a role in the crosstalk of visceral obesity with COVID-19 severity.

Recently, it was reported that obesity-related visceral adipose tissue, but not subcutaneous adipose tissue, shows hypermethylation and downregulation of the *ACE2* gene, that is mirrored in PBMCs and is restored after nutritional weight reduction therapy [17]. In that paper, the association was conjectured between lower *ACE2* expression and adverse cardiometabolic health indices, including type 2 diabetes and obesity status, through increased Angiotensin II [17]. In the present work, unfortunately we were unable to detect *ACE2* expression in PBCs, as it is at very low, practically undetectable levels [31]. However, we speculate that the observed increases in *ADAM17* expression in PBCs may mirror gene expression changes going on in visceral adipose tissue, where increases in *ADAM17* expression were to contribute to lower the levels of membrane bound ACE2 protein.

From our results, it is worth highlighting the large increase (7-fold) in the expression of the gene encoding the interferon-induced transmembrane (IFITM) protein 3 (*IFITM3*) in PBCs of patients with COVID-19, a gene upregulated soon after the infection of lung epithelial cells by SARS-CoV-2 [50]. IFITM proteins are important contributors to the immune response, which were initially thought to restrict virus entry by the endosomal route [19]. However, more recent work has suggested that IFITM3 may enhance SARS-CoV-2 fusion at the plasma membrane, with clear distinctions drawn between enhancement of viral infection at the plasma membrane and amphipathicity-based mechanisms used for endosomal SARS-CoV-2 entry restriction [51]. Furthermore, there is evidence that endogenous IFITM proteins may actually work as cofactors for efficient SARS-CoV-2 infection of clinically relevant human cell types [52]. Thus, from our results it can be hypothesized that increased IFITM3 expression in blood cells can contribute to the lymphopenia that is commonly observed in patients with COVID-19, and which was suggested could serve as a predictor for the prognosis of patients [53]. In fact, similar to that of *ADAM17* mRNA levels, the increase in *IFITM3* mRNA levels found in PBCs of COVID-19 patients was greater the higher the severity observed in the ultimate course of the disease, and maximal in patients with obesity (BMI > 30). A still poorly defined role of IFITM proteins in obesity development was suggested by the phenotype of mice lacking all five of the *Ifitm* genes, which develop an obese and metabolic syndrome phenotype related to hyperphagia, leptin resistance, and abnormal neuropeptide production, inflammatory status and microglia status in the hypothalamus [54]. Again, like results for *ADAM17*, a correlation between BMI and *IFITM3* gene expression in PBCs was found in the COVID-19 patients, but not in the controls, and it was stronger and of higher statistical significance when only the male patients were considered in the linear regression model. We suggest that the increased expression of both *ADAM17* and *IFITM3* plays an important role in the boosted pathogenesis of COVID-19 in male patients with obesity.

COVID-19 critical illness is driven by an exacerbated, pathological immune response (“cytokine storm”) against SARS-CoV-2 [55]. An “immune signature” for the early identification of patients more (or less) prone to develop a severe clinical condition is of high interest but yet to be clearly identified [55]. Among the proinflammatory interleukins and chemokines genes analyzed in the present work, *IL6*, *CXCL10* and *CXCL11* showed a differential expression (upregulation) in PBCs of COVID-19 patients on hospital admission relative to controls, the upregulation being proportional to the severity of the disease course for *CXCL11*. IL-6, CXCL10 and CXCL11 are among the blood circulating proteins found to be robustly associated with COVID-19 disease through a non-biased proteomics approach based on five clinical case-control studies [56]. Upregulations of IL-6 [43] and chemokine CXCL10 [55,57] signaling are key components of the SARS-CoV-2 induced cytokine storm. Circulating IL-6 has been related to COVID-19 severity in a number of studies and a comprehensive meta-analysis [28,58], and its elevated levels associate with respiratory failure in COVID-19 [59]. In the present work, upregulation of the expression of *IL6* in PBC did not show a clear relation with ulterior disease severity, yet there was a clear tendency to higher IL-6 circulating levels in those patients which experienced a more severe progression, who, in good concordance with previous reports [60], also had higher circulating levels of C-reactive protein (CRP). Among the proinflammatory signals analyzed for gene expression in PBCs of patients, only for the *IL6* gene an increased expression with increased BMI range was found. Considering that IL-6 produced by adipocytes and adipose tissue resident macrophages is one of the mediators of obesity-linked adipose tissue chronic inflammation [61], we suggest the increased *IL6* gene expression observed in the PBCs of COVID-19 patients with obesity may result from increased expression in excess adipose tissue, which we see reflected in PBCs (though not in the levels of IL-6 that reach systemic circulation).

Interferons (IFNs) are canonical mediators of antiviral signaling in the host that induce many essential components of the early host response to viral infection, including the group of IFITM proteins, among which the best characterized IFITM3 shows the greatest transcriptional response to type I interferon induction [54,62,63]. However, whether IFNs serve protective or detrimental functions in COVID-19 is a major unanswered question, with both protective and harmful effects being documented [64] and references therein. IFNs are a complex family of proteins, with 3 types and a total of 21 members in humans, and comprehensive studies indicate specialized IFN action in COVID-19 [65], and that the balance between different types of IFN [66], as well as the IFN landscape along the respiratory tract [64], impacts the severity of the disease.

Here, we show that the gene for type I IFNE is expressed in PBCs, where its basal level of expression (in the control subjects) and response to COVID-19 infection were sex-dependent. *IFNE* expression in PBC was higher in female than male control subjects and was increased by COVID-19 in the male patients only, to an extent that was related to both the severity course of the disease and the BMI range. A robust type I IFN response in severe COVID-19 patients has been described, which could exacerbate hyperinflammation through diverse mechanisms [67,68], but the induction of *IFNE* specifically and the radical differences depending on sex were unknown. Interestingly, IFNE is unusual among type I IFNs in that it is primarily constitutively expressed in the mucosal epithelium of the female reproductive tract, where it is hormonally regulated by sex hormones and it confers protection against sexually transmitted viral and bacterial infections [69]. Constitutive *IFNE* expression has been detected in some other mucosal epithelial sites, including lung in mice [69]. Because of its unusual expression and regulation in females, it has been speculated that IFNE may contribute to decreased mortality by SARS-CoV-2 infection in females [70]. Higher basal type 1 IFNs expression, including IFNE, may allow females to maintain a high T-cell level in the early stages of SARS-CoV-2 infection, leading to a milder evolution of COVID-19 [71]. In this context, increased expression of *IFNE* in male patients infected with SARS-CoV-2 could be viewed, therefore, as the turning on of a defense mechanism already in force by default in the females, but whose turning on in those circumstances contributes to worsen the disease. Thus, from the data herein, it can be proposed that the levels of expression of *IFNE*, as measured in PBCs, can be useful to predict different levels of severity outcome of COVID-19 in male patients, but not females. Differently to *IFNE*, *IFNG* (encoding type II IFNγ) was expressed at similar levels in PBCs in female and male control subjects, and its expression in PBCs was increased by COVID-19 in both sexes, to an extent not apparently related to the ulterior degree of disease severity or the patients’ BMI range. The differences may be ascribed to the different roles of type I and II interferons as first-line defense against viruses but having different functions [72].

*CCR2* (encoding CC chemokine receptor type 2) and *TYK2* (encoding tyrosine kinase 2) were identified as genetic mediators of COVID-19 critical illness in an unbiased search of genetic mechanisms behind this phenotype to identify causal variants [26]. Severe COVID-19 associated with CCR2 genetic variants that predict high expression of *CCR2* in lung tissue [26]. In other studies, direct associations between *CCR2* expression levels in PBCs and COVID-19 severity were detected [73,74]. Our results confirm *CCR2* gene expression in PBCs could be considered a prognosis marker of COVID-19 evolution, as it was found increased only in those patients that developed critical illness. CCR2 is the receptor for chemokine (C-C motif) ligand 2 (CCL2, also known as monocyte chemoattractant protein 1, MCP-1). The CCL2/CCR2 axis induces the recruitment of monocytes and macrophages towards sites of COVID-19 infection, and its hyperactivity results in hyperinflammation and organ damage [75]. Interestingly, in the present study, *CCR2* expression was somewhat higher, albeit non-significantly, in PBCs of patients with obesity, and BMI was predictive of *CCR2* gene expression levels in PBCs both in controls and patients, and more strongly in the male patients. Studies have shown an amelioration of the development of diet-induced obesity in male mice knockout for *Ccr2* [76,77], and of obesity-associated metabolic complications in genetically obese (db/db) male mice treated with a pharmacological inhibitor of CCR2 [78], suggesting CCR2 may influence the development of obesity.

*TYK2* encodes a Janus tyrosine kinase (JAK) family member that is an essential regulator of cytokine and type I IFN signaling [79]. Using Mendelian randomization, evidence was provided that high expression of *TYK2* is associated with life-threatening COVID-19 disease [26]. Additional studies have also highlighted the relevance of TYK2 in COVID-19 [80] and its involvement in COVID-19 severity [81]. In good concordance with these previous results, we found *TYK2* gene expression elevated in PBCs of COVID-19 patients on hospital admission, with a (non-significant) trend to higher levels in those that developed critical illness during hospitalization, and independent of the patient BMI range.

**Figure 6:**
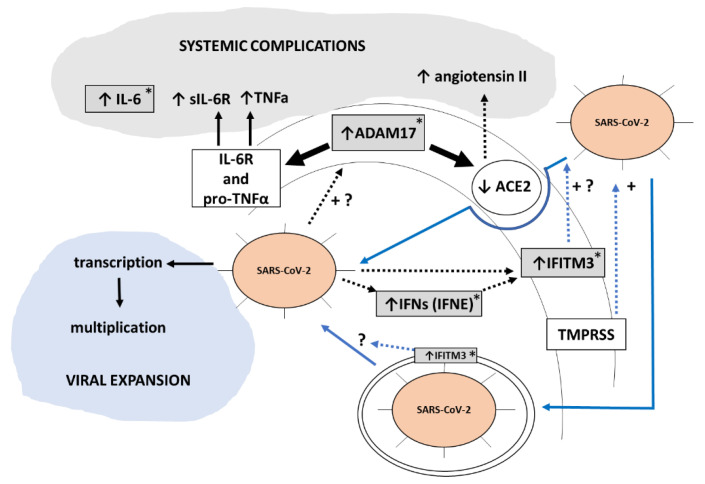
Scheme illustrating how gene expression changes observed in this work in PBCs used as sentinel cells could be related to and reflect COVID-19 severe evolution when extrapolate to clinically relevant tissues. Our results suggest that SARS-CoV-2 infection enhances ADAM17 gene expression and hence activity. Through increased proteolysis of its cell membrane substrates-Interleukin-6 receptor (IL-6R), pro-tumor necrosis factor alfa (pro-TNFa) and ACE2-increased ADAM17 activity will result in higher circulating levels of sIL-6R (the soluble form of the IL-6R), TNFa, and angiotensin II. These changes, together with increased circulating IL-6 levels, will contribute to systemic COVID-19 complications. Additionally, our results are indicative of a SARS-CoV-2 infection dependent induction of interferons (IFN) and interferon downstream targets such as IFITM3, which can promote SARS-CoV-2 infectivity and hence viral expansion according to previous reports [51]. Boxes in grey are changes observed in the current work in PBCs at the gene expression level. Blue lines represent the two main entry routes for SARS-CoV-2 into cells. Discontinuous lines indicate an indirect effect. +, enhancement effect; +?, suggested enhancement effect. Asterisks denote genes/proteins whose expression is elevated in COVID-19 patients with obesity, according to our results.

To summarize, in this work, a cluster of genes previously identified in lung tissue or other related tissues to be involved in the routes of SARS-CoV-2 entry into the human cells (*ADAM17* and *IFITM3*) or the host immunological response against the virus (*IL6*, *CXCL10*, *CXCL11*, *IFNE*, *IFNG*, *TYK2*) were found to be differentially expressed (upregulated) in PBCs from COVID-19 patients at the time of hospital admission as compared with non-infected controls. For *ADAM17* and *IFNE*, increased expression was observed mainly or only in the male patients. Importantly, for a number of these genes -*ADAM17*, *IFITM3*, *CXCL11*, *IFNE*, and also *CCR2*- expression levels in PBCs on hospital admission were higher in those patients that experienced a more serious evolution of the disease during hospitalization. However, more studies are needed to establish their relationship and/or causality. Moreover, importantly considering the need to further understand the obesity boosting effect on COVID-19 severe evolution, some of the genes analyzed -*ADAM17*, *IFITM3*, *IFNE*, *IL6*- were more highly expressed in PBCs of COVID-19 patients with obesity, and for three genes—*ADAM17*, *IFITM3* and *CCR2*- expression levels were dependent on the patients’ BMI, particularly in males. Thus, expression patterns of three genes (*IFITM3*, *ADAM17*, *IFNE*) in PBCs at the time of hospital admission marked both disease evolution severity and obesity status, especially in male patients. It should be mentioned as a limitation of the study that a stratification by BMI in the healthy volunteers included in the study was not established, due to the size of the sample. A scheme of the interrelations among the most interesting genes in this study with systemic complications and viral expansion in SARS-CoV-2 infection is shown in Figure 6. To be emphasized is that these results were obtained in minimally invasive samples, PBCs, that, therefore, could serve in clinical studies and the clinical practice to identify COVID-19 patients with different predictable severity evolution.

## Figures and Tables

**Table 1:** Anthropometric measurements in control subjects and COVID-19 patients. Data were analyzed considering all the individuals (women and men together), and stratified by COVID-19 severity (mild, severe, or critical) or by body mass index (BMI) range. Data are mean ± SEM. Statistics: differences between stratified groups were analyzed by one-way ANOVA followed by least significant difference (LSD) post-hoc test, a ≠ b ≠ c (*p* < 0.05). Student’s *t* test was used for single comparisons: *, COVID all vs Control, #, severe/critical vs mild COVID (*p* < 0.05).

	Control	COVID All	COVID by Severity			COVID by BMI		
			COVID (Mild)	COVID (Severe)	COVID (Critical)	COVID (BMI < 25)	COVID (BMI = 25–30)	COVID (BMI > 30)
Number of volunteers	47	73	33	24	16	20	32	21
Male/Female	15/32	43/30	15/18	15/9	13/3	9/11	19/13	15/6
	**Anthropometric Measurements**							
Age (years)	47.6 ± 1.8	58.3 ± 1.7 *	54.1 ± 2.6	61.8 ± 2.7	61.6 ± 3.0	57.9 ± 3.1	58.7 ± 2.8	58.0 ± 2.8
Weight (kg)	73.5 ± 2.7	77.5 ± 1.6	73.9 ± 2.4	78.1 ± 2.8	83.8 ± 3.3 #	64.7± 1.8 a	75.9 ± 1.4 b	91.9 ± 2.6 c
Height (cm)	166 ± 1	165 ± 2	164 ± 1.6	166 ± 1.4	163 ± 7	168 ± 1.9	165 ± 1.5	160 ± 5.5
BMI (kg/m^2^)	26.7 ± 0.1	28.1 ± 0.6	27.4 ± 0.9	28.3 ± 0.9	29.2 ± 1.2	22.7 ± 0.4 a	27.6 ± 0.2 b	33.9 ± 0.7 c

**Table 2:** Circulating parameters in COVID-19 patients. Data in each group are analyzed considering all the individuals (women and men together), and stratified by COVID-19 severity (mild, severe, or critical) or by body mass index (BMI) range. Data are mean ± SEM. Statistics: differences between stratified groups were analyzed by one-way ANOVA followed by least significant difference (LSD) post-hoc test, a ≠ b ≠ c (*p* < 0.05).

	COVID by Severity			COVID by BMI		
	COVID (Mild)	COVID (Severe)	COVID (Critical)	COVID (BMI < 25)	COVID (BMI = 25–30)	COVID (BMI > 30)
Number of volunteers	33	24	16	20	32	21
Male/Female	15/18	15/9	13/3	9/11	19/13	15/6
	**Circulating Parameters**					
Hemoglobin (g/dL)	13.8 ± 0.3	14.1 ± 0.3	13.9 ± 0.4	13.4 ± 0.4 a	13.8 ± 0.2 ab	14.6 ± 0.3 b
D-dimer (ng/mL)	184 ± 19	981 ± 333	561 ± 159	343 ± 123	529 ± 184	686 ± 279
Lactate dehydrogenase (U/L)	230 ± 17 a	345 ± 26 b	447 ± 37 c	277 ± 34	334 ± 27	340 ± 33
Bilirubin (mg/dL)	0.8 ± 0.1	0.9 ± 0.1	1.0± 0.1	1.0 ± 0.1 a	0.8 ± 0.1 b	0.9 ± 0.1 ab
GPT (U/L)	36.6 ± 8.1	54.0 ± 9.9	39.8 ± 4.9	31.3 ± 7.5	43.2 ± 5.7	54.3 ± 13.1
GOT (U/L)	29.5 ± 4.3 a	49.1 ±7.9 b	47.1 ± 5.5 ab	36.1 ± 7.0	39.4 ± 5.0	43.1 ± 7.2
GGT (U/L)	42.0 ± 6.7 a	81.5 ± 15.6 b	76.5 ± 15.6 ab	55.5 ± 16.2	61.5 ± 10.1	71.7± 12.7
Urea (mg/dL)	30.3 ± 1.8 a	40.0 ± 4.0 b	39.3 ± 3.1 b	36.8 ± 3.2	32.5 ± 2.0	38.9 ± 4.5
Glucose (mg/dL)	111 ± 12	130 ± 12	136 ± 12	108 ± 8	128 ± 13	130 ± 13
C-reactive protein (mg/dL)	4.39 ± 1.29 a	12.2 ± 1.74 b	12.3 ± 2.36 b	7.57 ± 1.73	8.84 ± 1.68	9.51 ± 2.12
Interleukin 6 (pg/mL)	49 ± 9.9	140 ± 47	304 ± 172	120 ± 49	160 ± 86	98 ± 43
Ferritin (ng/mL)	325 ± 93 a	984 ± 294 b	1002 ± 159 b	319 ± 80	887 ± 223	764 ± 186
Vitamin D (ng/mL)	14.7 ± 1.7	22.0 ± 3.8	14.5 ± 1.8	21.5 ± 4.1	14.9 ± 1.3	15.5 ± 2.6

## Supplementary Materials

The following supporting information can be downloaded at: https://www.mdpi.com/article/10.3390/biomedicines10082007/s1, Table S1: Nucleotide sequences of primers; Table S2: Detailed information for all the genes analyzed (mean, standard error of the mean (SEM), confidence interval, median, minimum, and maximum); Figure S1: Expression levels in PBCs of genes related to SARS-CoV-2 entry into cells in control subjects and COVID-19 patients (male and female) and separated according to COVID-19 severity and obesity in each sex.
