# Supplementary material for: Increased mRNA Levels of ADAM17, IFITM3, and IFNE in Peripheral Blood Cells Are Present in Patients with Obesity and May Predict Severe COVID-19 Evolution
Source: Biomedicines. 2022 Aug 18;10(8):2007. doi: 10.3390/biomedicines10082007 (PMC9406212; doi:10.3390/biomedicines10082007)
Supplement: Supplementary file 1 [file biomedicines-10-02007-s001.zip › biomedicines-1788636-SM.pdf]

Supplementary Table S1. Nucleotide sequences of primers.

Gene	Forward primer (5' to 3')	Reverse primer (5' to 3')	Amplicon size (pb)
ADAM17	CAACACAGATGGGGCCGAATA	ACTCGATGAACAAGCTCTTCAGG	216
IFITM3	GTCCAAACCTTCTTCTCTCCTGT	TCGCCAACCATCTTCCTGT	263
IFNE	AGCACTCATGGGACTGGAAG	CAGGTGCTGTAGTCCTGGTTT	126
IFNG	TTTGGGTTCTCTTGGCTGTT	CACTCTTTTGGATGCTCTGGT	233
CXCL8	GGCAGCCTTCCTGATTTCTG	CCAGACAGAGCTCTCTTCCA	206
CXCL9	GTGGTGTTCTTTTCCTCTTGG	ATAGTCCCTTGGTTGGTGCT	112
CXCL10	TCTGATTTGCTGCCTTATCTTTC	ACATCTCTTCTCACCCTTCTTTT	205
CXC11	TGTTCAAGGCTTCCCCATGT	CCTTGCTTGCTTCGATTTGGG	196
IL-6	ACCTTCCAAAGATGGCTGAA	CTGGCTTGTTCCTCACTACTCTC	151
IL-7	TCCCCTGATCCTTGTTCTGT	CCAATTTCTTTCATGCTGTCC	129
CCR2	AGCCACAAGCTGAACAGAGA	CCAGCATGTTGCCCACAAAA	250
TYK2	ATCACCCAGGCCTTCTACGA	CACCACCATCTTCCAAGCCA	192
EDN1	GGACATCATTTGGGTCAACA	CCCTGAGTTCTTTTCCTGCTT	185
TRIM27	GCGGAGACTAACGTGTCGTG	GGGCATCTGGTCCTGCTC	201

Supplementary Table S2. Detailed information for all the genes analyzed (mean, standard error of the mean (SEM), confidence interval, median, minimum, and maximum).

Gene	Sex	Control					COVID				
		Mean±SEM	Confidence interval (95%)	Median	Min.	Max.	Mean±SEM	Confidence interval (95%)	Median	Min.	Max.
**ADAM17**	*Male*	100±10.3	78.0-122	96.1	35.2	164	237±47.2	142-333	173	33.3	1957
	Female	133±14.9	103-164	107	2.9	399	147±12.4	121-172	142	6.5	295
**IFITM3**	*Male*	100±20.4	55.9-144	77.6	27.4	274	1247±395	449-2045	398	21.1	14775
	Female	165±23.7	117-214	109	47.9	521	1181±387	389-1973	398	46.4	10450
**IFNE**	*Male*	100±14.4	69.0-131	85.2	13.9	190	308±46.4	215-402	231	28.4	1069
	Female	302±88.8	121-483	126	11.2	2328	211±43.4	122-300	139	31.0	1160
**IFNG**	*Male*	100±25.5	45.3-155	70.3	11.1	382.5	134±29.1	75.1-193	89.4	2.0	843
	Female	67.5±15.5	35.8-99.1	40.0	10.5	449	131±25.2	80.0-183	88.1	16.0	565
**CXCL8**	*Male*	100±16.7	63.9-136	88.8	6.8	231	151±24.5	101-200	77.7	12.6	616
	Female	161±22.9	114-208	119	3.8	510	143±17.5	107-179	132	0.9	389
**CXCL9**	*Male*	100±19.9	56.7-143	95.9	14.7	248	228±70.6	84.0-372	87.6	12.7	2178
	Female	146±38.7	66.5-225	80.1	14.9	1069	106±13.5	78.5-134	108	8.0	288
**CXCL10**	*Male*	100±17.0	63.4-137	85.0	6.8	233	545±193	154-935	167	5.7	6185
	Female	180±34.4	110-250	1250	16.1	974.5	678±217	233-1122	198	21.9	4497
**CXCL11**	*Male*	100±20.1	56.2-144	67.6	6.8	266	414±123	165-664	185	6.5	4880
	Female	190±38.7	111-268	118	7.1	781	413±105	198-628	159	4.9	2039
**IL6**	*Male*	100±20.9	54.5-145	94.2	17.6	281	309±58.4	189-430	213	11.8	1290
	Female	174±30.1	113-236	122	22.2	691	261±44.8	169-353	241	7.1	1155
**IL7**	*Male*	100±16.7	63.9-136	99.3	23.5	265	122±18.8	83.7-160	102	8.4	636
	Female	97.6±20.9	55.1-140	66.7	11.5	661	78.1±10.6	56.3-99.8	65.3	1.1	284
**CCR2**	*Male*	100±4.7	89.8-110	108	64.8	121	147±27.7	90.7-203	114	14.9	1157
	Female	103±7.3	88.0-118	96.0	38.1	211	122±11.5	98.4-146	111	27.5	253
**TYK2**	*Male*	100± 14.9	67.3-133	92.9	18.4	210	272±39.2	192-352	207	67.6	1172
	Female	115±24.0	65.7-164	78.7	18.4	577.4	172±27.0	116-228	147	31.3	551
**EDN1**	*Male*	100±14.5	68.8-131	88.5	20.7	191	105±14.6	75.0-134	76.0	3.8	505
	Female	82.4±7.3	67.5-97.4	69.1	7.6	189	99.4±9.8	79.3-119	94.1	1.3	220

Genes relates to SARS-CoV-2 entry into cells

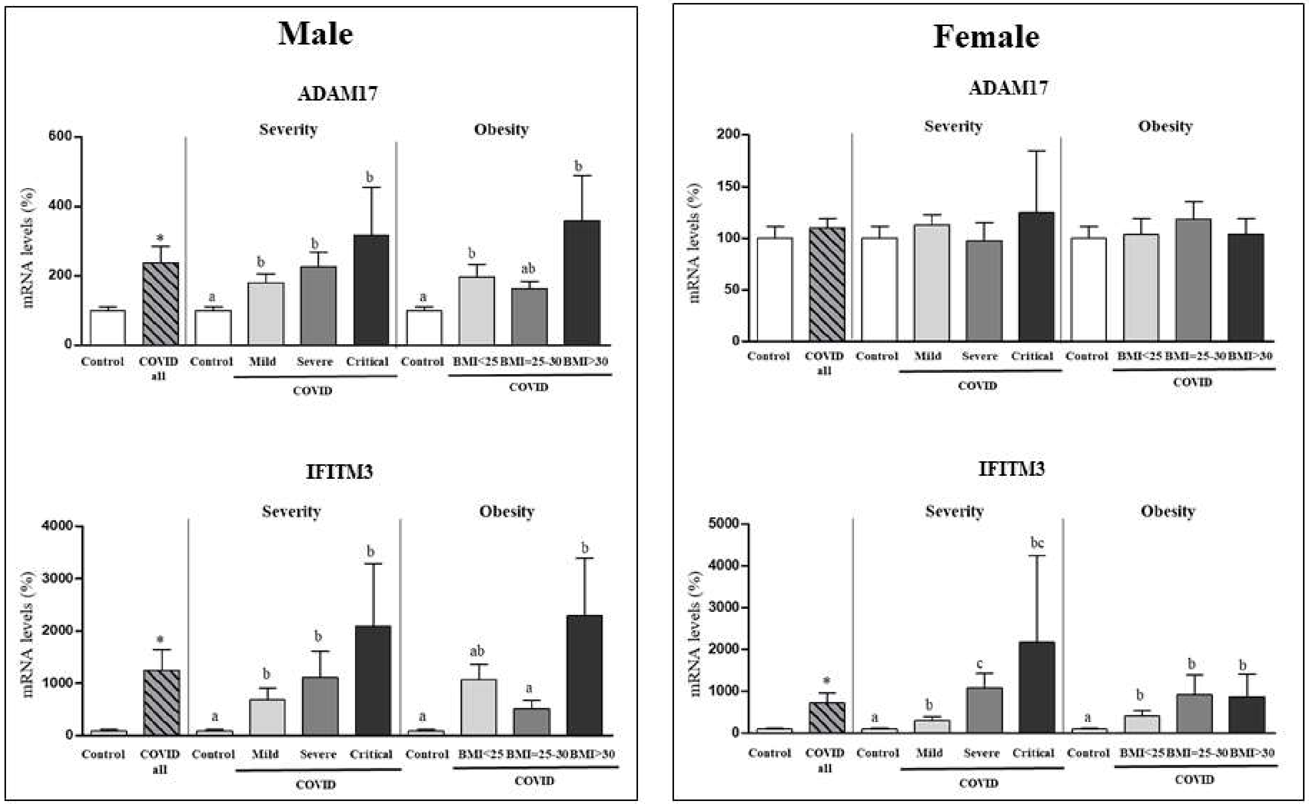

Supplementary Figure S1. Expression levels in PBCs of the indicated genes related to SARS-CoV-2 entry into cells in control subjects and COVID-19 patients (male and female) and separated according to COVID-19 severity and obesity in each sex. Statistics: differences between stratified groups were analyzed by one-way ANOVA followed by least significant difference (LSD) post-hoc test, a≠b≠c ($p<0.05$). Student's t test was used for single comparisons: *, COVID all vs Control.
